# Evaluation of antibacterial and acute oral toxicity of *Impatiens tinctoria* A. Rich root extracts

**DOI:** 10.1371/journal.pone.0255932

**Published:** 2021-08-11

**Authors:** Sileshi Degu, Abiy Abebe, Negero Gemeda, Adane Bitew

**Affiliations:** 1 Traditional and Modern Medicine Research Directorate, Ethiopian Public Health Institute, Addis Ababa, Ethiopia; 2 School of Medical Laboratory Science, College of Health Sciences, Addis Ababa University, Addis Ababa, Ethiopia; Thomas Jefferson University, UNITED STATES

## Abstract

The high prevalence of morbidity and mortality from bacterial infections, together with the growing threat of antibacterial resistance, necessitated the development of alternative new drugs from traditional medicine. In Ethiopia, *Impatiens tinctoria* A. Rich has been traditionally used for the treatment of fungal infections such as ringworms that cause tinea pedis and it have also different medical values. Scientific information on its biological activity against a broad range of bacteria and safety data is scant, compared to its folklore data. In this study, we evaluated antibacterial activities and acute oral toxicity of aqueous, ethanol and ethyl acetate root extracts of *Impatiens tinctoria* A. Rich. Aqueous, ethanol and ethyl acetate extracts of the plant were evaluated using agar hole diffusion and agar dilution methods. Biological activities of the plant extracts were expressed as a zone of inhibition diameter, minimum inhibitory concentration (mg/ml), and minimum bactericidal concentration (mg/ml). The safety studies were performed by oral acute toxicity study according to the organization of economic cooperation and development test Guidelines 420.Gram-positive bacteria were more susceptible to the extracts compared to gram-negative bacteria, especially against *S*. *aureus* and *S*. *epidermidis*, which are commonly found in the skin. Ethyl acetate extract was more potent than ethanol and aqueous extracts. The 50% lethal dose (LD50) of tested mice was above 9600 mg/kg. This study provides a scientific basis for the antibacterial activity of the root extracts of I. *tinctoria* A. Rich, where, the ethyl acetate extract showed the most promising activity. Therefore, the antibacterial potential and practical non-toxicity of the study plant extracts suggested the possibility of using it for the development of antimicrobial drugs by further studying the plant in different directions.

## Introduction

Infectious diseases are the world’s leading cause of premature deaths, killing almost13.4 million people per year. The World Health Organization (WHO) forecasts 13 million deaths attributed to this cause in 2050 [1,2]. Infections due to a variety of bacterial etiologic agents become common and are taking the big share of the burden [1]. Severe infections, including sepsis, meningitis, and pneumonia, are estimated to cause approximately one-third of the 2.6 million neonatal deaths globally, most of which are in less affluent regions of our planet [1]. Similarly, in Ethiopia, infectious diseases are the top five leading causes of premature mortality [3].

There is also an alarming increase in the incidence of new and reemerging infectious diseases, some of which do not have drugs that act against them [4,5]. For instance, over the past 40 years, a minimum of 50 emerging infectious agents have been identified across the globe; approximately 10% of them are bacterial agents [5]. Additionally, drug resistance has been commonly reported worldwide [6]. For example, the development of methicillin resistance has decreased the usefulness of this antibiotic in treating serious staphylococcal infections within the community and hospitalized patients [6]. Presently, approximately 60,000 people in Europe and the United States die each year due to serious infections caused by antimicrobial-resistant bacteria [7]. The problem is also high in Ethiopia, as indicated by few studies [8–11].

Despite such problems, medicinal plants have been used since ancient times to treat various diseases. They are the basis for most traditional healing practices in which approximately 4.3 billion people of the world’s population use herbal medicines for some aspect of primary healthcare [12]. Surveys carried out in developed countries such as Germany and Canada tend to show that no less than 70% of their population has used herbal remedies at least once, reaching 80% when we come to the emerging world [13]. Traditional remedies are the most important and sometimes the only source of therapeutics for nearly 80% of the Ethiopian population, and 95% of the preparations are of plant origin [14].

Therefore, scientific studies must be conducted on traditional medicinal plants to develop new, effective and safe antimicrobial drugs. Locally, Ethiopian women chop or mash the inside of the roots of I. *tinctoria* A. Rich into a paste to dye the palms and nails of the hands and feet as a beauty treatment to control fungal infections and to toughen the skin [15,16]. The root decoction is also drunk against abdominal pains and as a purgative. The stem is chewed to treat mouth and throat diseases [17]. Besides, the folk information, to our knowledge, there is no or limited scientific evidence on the antimicrobial activity of this plant against broad range of bacteria. Because of this, this study aimed to scientifically justify the antimicrobial potential of this medicinal plant’s root extract against selected bacteria. Moreover, assuming the presence of minimal absorbance through the skin during traditional application, acute oral toxicity was evaluated to identify the range of treatment doses that could be used and the possible clinical signs elicited by this plant extract.

## Materials and methods

### Herbal material collection and preparation

The whole plant material of *Impatiens tinctoria* A. Rich was collected from Butajira, Southern Nations and Nationalities Region, Ethiopia and identified by a botanist. The collected root part was washed with clean water, cut, and dried at room temperature using a milling machine, and the plant cutlets were milled to powder. The powder was weighed using an electronic weighing balance, packed in polyethylene bags to avoid entrance of air and any other contaminant and stored in a closed container with proper labeling for further extraction processes.

### Extraction

The extraction was performed by mixing the root powder and extraction solvents (distilled water, ethanol and ethyl acetate) with a proportion of 1 gram of powder and 20 ml solvents. After thoroughly mixing, the samples were macerated in a rotaryshaker (VWR DS-500, USA)at 100 rpm for 24 hours and filtered through Whatman filter paper, followed by concentration under reduced pressure (vacuum) by a rotary evaporator(R-200 Buchi, Switzerland)at 40°C to obtain the extract. These concentrated extracts were kept in a water bath, settedat 40°C, to avoid the remaining organic solvent and water [18,19].

### Microorganisms

The antibacterial activity of the root extracts of the study plant was evaluated on 13 standard strains (American Type Culture Collection (ATCC)) or clinically isolated bacteria. The tested bacterial strains were *S*. *aureus* (ATCC 25923), Methicillin-resistant Staphylococcus aureus(MRSA) (clinical isolate), *S*. *epidermidis* (ATCC 12228), *S*. *pyogenes* (ATCC 19615), *Streptococcus agalactiae* (*S*. *agalactiae* (ATCC 12386)), *E*. *faecalis* (ATCC 29212), *E*. *coli* (ATCC 25922), *S*. *typhimurium* (ATCC 13311), *Shigella flexneri (S*. *flexneri*(ATCC 12022)),*Shigella sonnei (S*. *sonnei*(ATCC25931)), *P*. *aeruginosa* (ATCC 27853), *K*. *pneumoniae* (ATCC 700603) and *Proteus mirabilis (P*. *mirabilis* (ATCC 35659)).These microorganisms were maintained in the laboratory of microbiology in the Traditional and Modern Medicine Research Directorate(TMMRD) of Ethiopian Public Health Institute(EPHI) on Triptosoya + 20% glycerol broth at -78°C.

### Inoculum preparation

All strains were refreshed for the actual test in Petri dishes containing nutrient agar, except *S*. *pyogenes* (ATCC 19615) and *S*. *agalactiae* (ATCC 12386), which were grown on 5% sheep blood nutrient agar, by incubation for 18–24 hours at 37°C. The grown bacteria few inoculums were harvested using 5 ml of nutrient broth, and its absorbance was adjusted at 625 nm and diluted to attain a viable cell count of 10^7^ CFU/ml using a spectrophotometer [20,21].

### Antimicrobial assay

#### Screening antimicrobial activity

The agarhole diffusion method was used to evaluate the antimicrobial activity of the extract. The agar plate surface was inoculated by spreading a volume of the microbial inoculums, taken from adjusted suspensions, over the entire agar surface. Then, a hole with a diameter of 8 mm has punched aseptically with a sterile cork borer or tip, and a volume (100μl) of the antimicrobial agent or extract solution at the desired concentration (100 mg/ml, 200 mg/ml and 400 mg/ml) was introduced into the well. Then, the plates were incubated under suitable conditions (at 37°C for 18–24 hours). The antimicrobial agent diffuses in the agar medium and inhibits the growth of the microbial strain tested. The presence of inhibition zones was measured by a ruler and considered an indication for antimicrobial activity [22].

#### Minimum inhibitory concentration

All tested extracts were manipulated to determine their minimum inhibitory concentration (MIC) using the agar dilution method by preparing different concentrations of extracts (from 64 mg/ml to 0.0625 mg/ml) through two-fold serial dilution. Then, the prepared concentrations of the extracts were incorporated into an agar medium (molten agar medium), followed by the inoculation of defined microbial inoculums onto the agar plate surface. The incubation time was similar to that of the screening method. The MIC was considered the lowest concentration that inhibited the growth of the respective bacteria under suitable incubation conditions and was expressed in mg/ml [23].

#### Determination of minimum bactericidal concentration

Streaks were taken from all concentrations of the plant extract plates exhibiting invisible growth (from the inhibition zone of MIC plates) and subculture onto the appropriate media plate. The plates were incubated under suitable conditions depending upon the test microorganism (at 37°C for 18–24 hours). Then, bacterial growth corresponding to extract concentrations was examined. The minimum bactericidal concentration (MBC) was taken as the concentration of plant extract that did not exhibit any bacterial growth on the freshly inoculated agar plates. All assays were performed in triplicate [24].

### Oral acute toxicity

An acute toxicity study was performed according to the Organization of Economic Cooperation and Development (OECD) test Guidelines 420 (Acute Oral toxicity–Fixed dose procedure) with slight modification. Healthy young adult, nulliparous and non-pregnant female albino mice were used. The testing animals were randomly selected from 8- to 12-week-old mice, marked to permit individual identification, and kept in their cages for 5 days before dosing to allow for acclimatization to the laboratory conditions. The animals were fasted 3–4 hours (food withdrawn but not water) before dosing, after which the animals were weighed to determine the fasted body weight. Each animal, at the commencement of its dosing, was 25–33 gm weight [25].

The starting dose was 300 mg/kg, which was increased by bifold until 9600 mg/kg. Five animals were used for each dose. Treatment of animals at the next dose was delayed until confident survival of the previously dosed animals was assured. The extract was calculated according to the body weight and dissolved in a consideration of the administered volume not exceeding 1 ml/100 g of mouse body weight. Then, the solvent alone for the control groups and the diluted extract for the treated groups were administered oral gavage. After administration, each mouse was closely observed for the first 30 minutes, hourly during the first six hours, two hours during the first 24 hours, and daily for a total of 14 days. All observations, such as changes in breathing, alertness, restlessness, diarrhea, behavioral pattern, mortality and consumption of food and water, were systematically recorded. Moreover, the body weight was measured on the initial day and thepost-treatment days 7 and 14 [25].

### Data analysis and interpretation

The extracted data were examined for completeness and checked for consistency. Then, the data were entered into an Excel spreadsheet, exported to Minitab 16 software and analyzed. The significant differences in the antibacterial activity of crude extracts on each bacteria and the effect of each extract on the bodyweight of the albino mice were carried out by employing one-way analysis of variance (ANOVA) followed by Tukey’s multiple comparison tests. The experimental data are expressed as the mean ± standard deviation (SD). The result was considered statistically significant at *P<0*.*05*.

### Ethics approval and consent to participate

The Departmental Research and Ethics Review Committee of the Department of Medical Laboratory Sciences, College of Health Sciences, Addis Ababa University approved the study protocol by giving protocol number DRERC/392/19/MLS. Laboratory animals were managed scientifically according to the international guidelines of the care and use of laboratory animals.

## Results

### Antibacterial activity screening by the agar-holemethod

The antibacterial activity of aqueous, ethanol and ethyl acetate extracts of the roots of *I*.*tinctoria* A. Rich was screened against selected bacteria. A total of 13 bacteria were assessed by agar well diffusion assay at concentrations of 100, 200, and 400 mg/ml for each extract in triplicate.

#### Antibacterial activity against gram-positive bacteria

**Fig 1:**
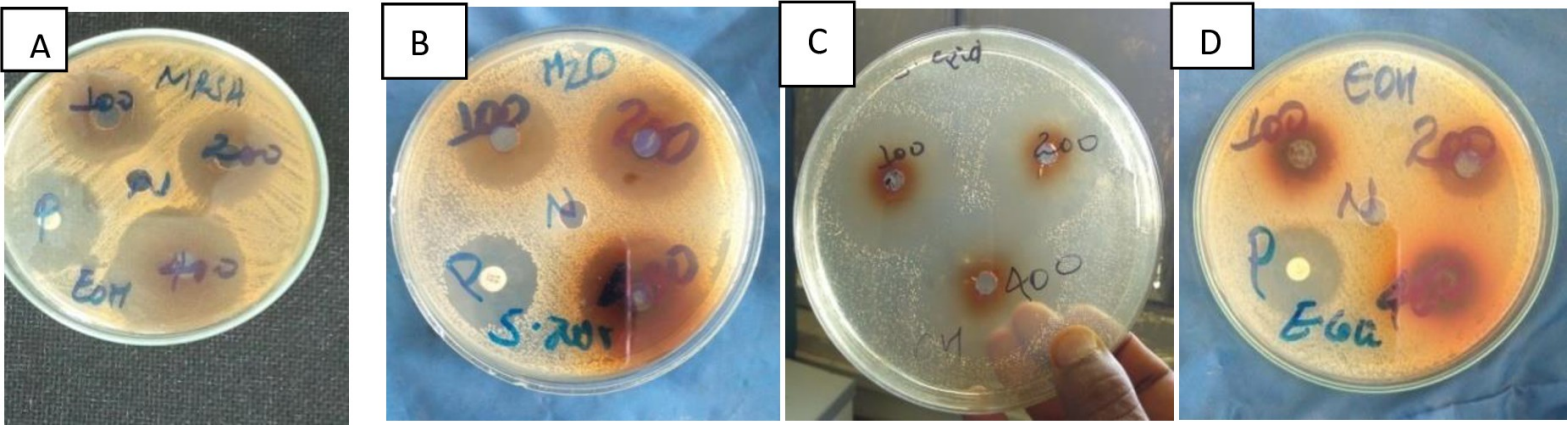
Example of antimicrobial activity of plant extracts using agar diffusion: MRSA by ethanol extract (A), S.*aureus* by aqueous extract (B), S.*epidermidis*by ethanol extract*(*C), E.*coli*by ethanol extract (D) at 100,200,400mg/ml concentrations.

The average zone of inhibition formed by all tested concentrations of ethanol and ethyl acetate extracts against *S*. *aureus* was better compared to the aqueous extract, significantly different at *P<0*.*05*. Each extract produced a notable inhibition zone against MRSA at all concentrations. *S*. *epidermidis* produced the largest average zone of inhibition when compared to the other tested gram-positive bacteria, in which the inhibition zone against this bacterium ranged from 38 mm by 100 mg/ml aqueous extract to 44 mm by 400 mg/ml ethyl acetate extract (Fig 1). Comparisons of the mean growth inhibition zones for *S*. *aureus*at 100, 200 and 400 mg/ml concentrations of the three tested extracts showed no significant differences (*P* < 0.05).This indicates the absence of a concentration-dependent inhibition difference, which was also observed on MRSA and *S*. *epidermidis*.

All extracts showed a lower inhibition zone against the two Streptococci bacteria (*S*.*pyogen* and *S*.*agalactiae*) than the other tested gram-positive bacteria at each corresponding concentration. Erythromycin (15 μg) showed a better inhibition diameter against the two tested streptococci than the three extracts at all concentrations that were significantly different at p<0.05. In an extract type-dependent manner, better inhibition against *S*. *pyogen* and *S*. *agalactiae*was obtained by ethyl acetate extract followed by ethanol extract. The smallest inhibition diameter of 17 mm for 100 mg/ml aqueous extract against *S*.*agalactiae* was recorded when compared with all tested gram-positive bacteria.

The three extracts showed better inhibition of *E*. *faecalis* at 400 mg/ml than at 100 mg/ml and 200 mg/ml, with a significant difference at P<0.05. The effect of vancomycin (30 μg) on this bacterium revealed less inhibition of all extracts and doses, with a significant difference at P<0.05, except for the aqueous extract at 100 mg/ml concentration. Similarly, erythromycin (15 μg) that produced a 21 mm inhibition zonewas greater than the inhibition of aqueous extract at 100 mg/ml concentration and less than or equal to other extracts at all concentrations. The potency of almost all extracts on this bacterium was better than that of *S*. *pyogenes* and *S*. *agalactiae* but less than that of *S*. *epidermidis*, *S*. *aureus* and MRSA (Table 1).

**Table 1:** Inhibition zone diameter measurement (mm) of crude extracts of *I*. *tinctoria* A. Rich roots against gram-positive bacteria.

Different solvent extracts (mg/ml)		Inhibition Zone Diameter (mm), including hole diameter (8 mm)					
		*S*. *aureus*	MRSA	*S*. *epid*	*S*. *pyogen*	*S*.*agal*	*E*. *faecalis*
Aqueous extract	100	26±0.6^d^	27±1.0^d^	38±1.0^b^	18±0.0^f^	17±0.6^h^	19±1.0^de^
	200	27±0.6^cd^	28±0.7^cd^	41±1.0^ab^	22±0.6^e^	18±0.0^gh^	22±0.6^cd^
	400	29±1.2^bc^	29±0.6^cd^	42±1.5^ab^	24±1.0^cd^	21±0.6^cde^	28±1.5^ab^
Ethanol extract	100	35±1.0^a^	30±0.7^bcd^	41±1.7^ab^	22±0.6^de^	19± 0.0^fgh^	21±1.5^cde^
	200	36±0.6^a^	30±0.6^bcd^	42 ±1.0^a^	23±0.6^de^	20±1.5^efg^	28±0.6^ab^
	400	37±0.6^a^	32±1.5^bc^	43±1.0^a^	24±1.0^cd^	22±1.2^cde^	29 ±1.2^a^
Ethyl acetate extract	100	36±0.6^a^	30±1.7^bcd^	41±0.6^ab^	26±0.6^b^	22±0.6^bcd^	24±0.6^bc^
	200	36±1.0^a^	33±2.7^ab^	42±1.0^a^	27±0.6^b^	23±1.0^bc^	28±0.6^ab^
	400	37±1.0^a^	36±1.2^a^	44±1.5^a^	26±1.0^b^	24±1.0^b^	30±1.5^a^
Distilled water	-	8±0.0^f^	8±0.0^f^	8±0.0^e^	8±0.0^g^	8±0.0^i^	8±0.0^f^
5% tween 80	-	8±0.0^f^	8±0.0^f^	8±0.0^e^	8±0.0^g^	8±0.0^i^	8±0.0^f^
Vancomycin	30μg	21±1.5^e^	21±1.5^e^	22±2.1^d^	22±1.0^de^	21±0.6^def^	19±1.2^e^
Erythromycin	15μg	38±2.1^b^	32±2.1^bc^	32±2.1^c^	30±1.5^a^	31±1.0^a^	21±2.3^cde^

**Keynotes**: Values are expressed as the mean ± SD (n = 3), 8±0.0 = no inhibition (hole diameter), “Means” that do not share a superscript letter are significantly different (only column wise) at P<0.05. *S*. *epid- S*. *epidermidis*, *S*. *agal- S*. *agalactiae*.

#### Antibacterial activity against gram-negative bacteria

*S*. *flexneri*, *S*. *soni and P*. *mirabilus* showed the highest inhibition zone among all gram-negative bacteria at 400 mg/ml ethyl acetate extract with an inhibition zone of 23 mm compared to other gram-negative bacteria. *K*. *pneumoniae* measured the smallest inhibition zone of 17 mm at this concentration and extract type. However, the growth of this bacterium did not show any inhibition by 100 mg/ml aqueous extract.

The positive control, ciprofloxacin (5 μg/ml), showed significantly higher inhibition of the growth of all gram-negative bacteria compared to all tested extracts at all concentrations (*P*<0.05). On the other hand, among the extract types, the aqueous extract showed significantly lower inhibition activity against the tested gram-negative bacteria (*P*<0.05). Moreover, no statistically significant differences in inhibition were observed against *E*. *coli*, *S*. *typhimurium*, *P*. *aeroginosa*, *K*. *pneumoniae* and *P*. *mirabilus* at a concentration of 100 mg/ml of aqueous extract compared to the negative control (distilled water and 5% Tween 80) (*P*<0.05). Table 2 shows the inhibition zone diameter and association of the seven tested gram-negative bacteria used to assess the antibacterial activity of the extracts.

**Table 2:** Inhibition zone diameter measurement (mm) of crude extracts of*I*.*tinctoria* A. Rich roots against gram-negative bacteria.

Extract Type	Conc.	Inhibition Zone Diameter (mm), including hole diameter (8 mm)						
		*E*.*coli*	*S*.*typhim*	*S*.*flexneri*	*S*.*sonnei*	*P*.*aerog*	*K*.*pneum*	*P*.*mirabi*
Aqueous extract	100	9±0.6^e^	9±0.0^ef^	11±0.6^h^	11±0.6^g^	11±1.0^f^	8±0.0^f^	9±0.6^g^
	200	12±0.6^d^	12±0.6^e^	13 ±0.6^g^	13±1.0^fg^	14±0.6^ef^	9±0.0^f^	12±0.6^f^
	400	15±0.0^c^	15±0.6^d^	17±0.6^f^	15±0.0^ef^	16±1.0^de^	12±1.0^e^	15±0.6^e^
Ethanol extract	100	16±0.6^c^	17±0.6^cd^	16± 1.0^f^	15±0.6ef	18±1.2^cd^	14±0.6^de^	19±0.6^cd^
	200	19±0.6^b^	19±1.0^bc^	17±0.6^ef^	15±0.6^ef^	19±1.5^bc^	15±0.0^cd^	21±0.6^bc^
	400	21±0.6^b^	21±1.5^b^	19±0.6^de^	17±1.2^df^	20±1.2^bc^	17±0.6^bc^	23±0.6^b^
Ethyl acetate extract	100	16±0.6^c^	17±0.6^cd^	20±0.0^cd^	19±1.0^cd^	17±1.2^cd^	15±0.6^cd^	19±0.6^d^
	200	19±1.5^b^	19±1.5^bc^	21±0.6^bc^	21±1.5^bc^	19±1.5^bc^	16±1.5^bcd^	20±1.5^cd^
	400	21±1.0^b^	21±1.7^b^	23±0.6^b^	23±2.3b	22±0.6^b^	17±1.2^b^	23±1.5^b^
DW	-	8±0.0^e^	8±0.0^f^	8±0.0^i^	8±0.0h	8±0.0^g^	8±0.0^f^	8±0.0^g^
5% T80	-	8±0.0^e^	8±0.0^f^	8±0.0^i^	8±0.0^h^	8±0.0^g^	8±0.0^f^	8±0.0^g^
Cipro	5μg	31±0.6^a^	34±0.6^a^	32±0.6^a^	31±0.0^a^	29±0.6^a^	23±0.6^a^	32±0.6^a^

**Keynotes**: Values are expressed as the mean ± SD (n = 3), 8±0.0 = no inhibition (hole diameter). Means that do not share a superscript letter are significantly different (only column wise) at *P*<0.05. Conc-Concentration, Cipro-Ciprofloxacillin as positive control, DW- Distilled Water as negative control 1, *P*.*aero-P*. *aeruginosa*, *S*.*typhim*-*S*. *typhimurium*, T80-Tween 80 as negative control 2.

### Minimum inhibitory concentration and minimum bactericidal concentration of the extracts

Each tested microorganism was examined, starting from a high concentration of 64 mg/ml by descending with serial bi-fold dilution to 0.0625 mg/ml, to determine the MIC value. Based on the study, the MIC value of the extracts was in agreement with its preliminary antimicrobial activity screening (on-well method) against most of the microorganisms. The ethyl acetate extract of the plant was more potent against all organisms than ethanol and aqueous extracts, which was also supported by the MBC value. *S*. *aureus* and *S*. *epidermidis* were inhibited at lower concentrations by ethanol extract than aqueous extract. For other microorganisms, the two extracts showed similar MIC values.

*S*.*epidermidis* was the most susceptible bacteria, with MIC values of 0.7±0.3 mg/ml,1.0±0.0 mg/ml, and 2.0±0.3 mg/ml for ethyl acetate, ethanol and aqueous extracts, respectively. The low antimicrobial activity was recorded by *E*. *faecalis*, with the highest MIC value of 8.0±0.0 mg/ml by ethyl acetate extract and 16.0±0.0 mg/ml by both ethanol and aqueous extracts compared to other gram-positive bacteria. Of the gram-negative bacteria, *S*. *typhimurium*, *S*. *sonnei* and *P*. *mirabilus* were the most susceptible bacteria and showed similar MIC values as *E*. *faecalis* in the three extracts. The lowest antimicrobial activities were recorded by *P*. *aeroginosa* and *K*. *pneumonia*, with MIC values above 64 mg/ml for aqueous and ethanol extracts and 16 mg/ml for ethyl acetate extracts. These values were in agreement with MBC values in which E. *coli*, *K*. *puemoniae*and *P*. *aeroginosa* MBC values were above 64 mg/ml by ethanol and aqueous extracts and were ≤64 mg/ml in the case of ethyl acetate extract. The minimum MBC value among all bacteria was 4.0±0.0 mg/ml, which was obtained by ethyl acetate extract against *S*. *epidermidis* (Table 3).

**Table 3:** MIC and MBC values of the tested microorganisms.

Microorganisms	Aqueous extract		Ethanol extract		Ethyl acetate extract		Positive control	
	MIC	MBC	MIC	MBC	MIC	MBC	MIC	MBC
*S*.*aureus*	8.o±0.0	32.0±0.0	2±0.0	16±0.0	0.8±0.3	8±0.0	0.5±0.0	1.0±0.0
MRSA	8.0±0.0	32.0±0.0	8±0.0	32±0.0	4±0.0	8±0.0	0.5±0.0	4.0±0.0
*S*.*epidermidis*	2.0±0.0	16.0±0.0	1±0.0	16±0.0	0.7±0.3	4±0.0	0.25±0.0	0.25±0.0
*S*.*pyogenes*	4.0±0.0	4±0.0	16.0±0.0	16±0.0	2±0.0	8±0.0	0.5±0.0	4±0.0
*S*.*agalactiae*	4.0±0.0	16.0±0.0	4±0.0	16±0.0	2±0.0	8±0.0	0.5±0.0	1±0.0
*E*.*faecalis*	16.0±0.0	32.0±0.0	16.0±0.0	32±0.0	8.0±0.0	16±0.0	0.5±0.0	4±0.0
*E*.*coli*	32±0.0	>64±0.0	32±0.0	>64±0.0	8±0.0	16±0.0	0.5±0.0	0.5±0.0
*S*.*typhimurium*	16±0.0	32±0.0	16±0.0	32±0.0	8±0.0	16±0.0	0.08±0.04	0.5±0.0
*S*.*flexneri*	16±0.0	32±0.0	16±0.0	32±0.0	8±0.0	16±0.0	0.08±0.04	0.5±0.0
*S*.*sonnei*	16±0.0	32±0.0	16±0.0	32±0.0	8±0.0	16±0.0	0.5±0.0	0.5±0.0
*P*.*aeroginosa*	>64±0.0	>64±0.0	>64±0.0	>64±0.0	16±0.0	32±0.0	1.0±0.0	2±0.0
*K*.*pneumonia*	>64±0.0	>64±0.0	>64±0.0	>64±0.0	16±0.0	32±0.0	1.0±0.0	4±0.0
*P*.*mirabilus*	16±0.0	32±0.0	16±0.0	32±0.0	8±0.0	16±0.0	0.5±0.0	0.5±0.0

**Keynotes:** Erythromycin and ciprofloxaciline are positive control drugs for gram-positive bacteria and gram-negative bacteria, respectively. The MIC and MBC values are expressed in mg/ml for extracts and in μg/ml for positive controls.

### Acute oral toxicity study

#### Behavioral pattern and LD50

The results of an acute oral toxicity study showed that the aqueous extracts of the plant appeared to be safe up to the dose of 9600 mg/kg. Testing parameters of restlessness, touch response, pain response, urination, skin color, fur erection, and food and water intake were assessed (Table 4). Drowsiness and erection of fur were observed at doses of 4800 and 9600 mg/kg. Nevertheless, other groups did not show any sign of toxicity. Generally, the study revealed the absence of signs of toxicity for most of the set parameters and the absence of mouse death records up to the 14^th^ day. Therefore, the LD50 of the extract might be considered to be greater than 9600 mg/kg.

**Table 4:** General appearance and behavioral observations of acute toxicity study for control and treated groups.

Observation	Dose of extracts in mg/kg						
	Control	300	600	1200	2400	4800	9600
Food intake	Normal	Normal	Normal	Normal	Normal	Normal	Normal
Water intake	Normal	Normal	Normal	Normal	Normal	Normal	Normal
Diarrhea	Not seen	Not seen	Not seen	Not seen	Not seen	Not seen	Not seen
Urination	Normal	Normal	Normal	Normal	Normal	Normal	Normal
Breathing	Normal	Normal	Normal	Normal	Normal	Normal	Normal
Skin color	Normal	Normal	Normal	Normal	Normal	Normal	Normal
Drowsiness	Not seen	Not seen	Not seen	Not seen	Not seen	present	Present
Hypersensitivity	Not seen	Not seen	Not seen	Not seen	Not seen	Not seen	Not seen
Erection of fur	Not seen	Not seen	Not seen	Not seen	Not seen	present	Present
Sedation	Not seen	Not seen	Not seen	Not seen	Not seen	Not seen	Not seen
Death	Alive	Alive	Alive	Alive	Alive	Alive	Alive

Keynotes: n = 5,“present” means at least 1 out of the 5 mice showed the symptom, “not seen/normal” means no mice showed the symptom.

#### Bodyweight

**Fig 2:**
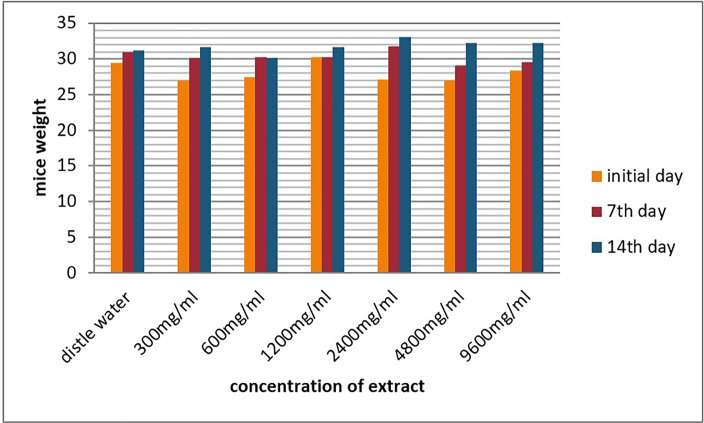
The effect of the aqueous extract of I. *tinctoria* A. Rich roots on the bodyweight of mice on different days.

The weekly body weights of the six groups were measured on the initial day and on the 7th and 14^th^ days, as displayed in Fig 2. At these days, all treated groups did not show any statistically significant differences in body weight compared with the control groups (*p<0*.*05*).

## Discussion

### Antibacterial activity

The inhibition zone of the most susceptible bacteria (*S*. *aureus*, *S*. *epidermidis* and MRSA) in the hole method assay did not show any significant difference (p<0.05) at the tested concentrations of 100 mg/ml, 200 mg/ml and 400 mg/ml for most tested extracts. Therefore, these bacteria have resulted in similar susceptibility at both the lowest and highest tested extract concentrations in this method. These observations could be explained as the effect of these concentrations might be the maximal efficacy portion on the dose-response curve, where the steepest portionmight be below 100 mg/ml concentration with the assumption that the dose-response curve is sigmoidal. From this, the minimum effective dose might be≤100 mg/ml. The remaining tested gram-positive bacteria *S*. *pyogenes*, *S*. *agalactiae* and *E*. *faecalis* also did not show any significant difference inhibition at 200 mg/ml and 400 mg/ml concentrations for ethanol and ethyl acetate extracts, which was a similar scenario with most gram-negative bacteria.

The antibacterial activity of the extracts against MRSA resulted in the highest inhibition zone and lowest MIC and MBC values of 36 mm, 4 mg/ml and 8 mg/ml, respectively. All extracts at all concentrations (100 mg/ml, 200 mg/ml and 400 mg/ml) showed better antibacterial activity than vancomycin (30 μg) on the hole method, with a statistically significant difference at p<0.05. This might be due to the ability of the extracts to inhibit penicillin-binding proteins of the bacteria that are involved in the synthesis of peptidoglycan, which is impossible by the antibiotic methicillin [26]. Therefore, it could be a good alternative as a natural product, as we are now in a situation where, in some cases, the glycopeptide antibiotic vancomycin is the only option for antimicrobial therapy even though its non-susceptibility in *S*. *aureus* is on the increase [6,26].

The hole method zone of inhibition was in line with the MBC and MIC value concentrations for most of the tested microorganisms except *S*. *pyogenes* and *S*. *agalactiae*, which might suggest the consistency of the testing methods. The inconsistency of the two organisms might be due to the usage of 5% sheep blood muller-hinton agar. The sheep blood might, to some extent, decrease the looseness of the media that lead to a weak diffusion of extracts than the pure muller-hinton agar that is used for other bacteria. On the other hand, these two bacteria might be susceptible to large molecules or hydrophobic molecules of the extract constituents, which did not diffuse easily, as other studies support it [27]. These might be the reasons that the two organisms record better MIC and MBC values than those bacteria that had longer inhibition zones. For instance, *S*. *agalactiae* and *E*. *faecalis* on 400 mg/ml ethyl acetate extract showed inhibition zones of 24 mm and 30 mm, respectively (significantly different at p<0.05). This value was inversed to the *S*. *agalactiae* records of 2 mg/ml and 8 mg/ml, whereas *E*. *faecalis* records 8 mg/ml and 16 mg/ml MIC and MBC values, respectively.

As observed from the inhibition zone, MIC and MBC values of the extracts the study plant also showed antibacterial activity against gram-negative bacteria in an extraction solvent-dependent manner. Of the extracts, ethyl acetate extract showed better antibacterial activity against all gram-negative bacteria. For example, *K*.*peumoniae* and *P*.*aeroginosa* had>64 mg/ml of both MIC and MBC on water and ethanol extracts, whereas ethyl acetate extract had 16 mg/ml MIC and 32 mg/ml MBC, which was a great difference between them. This notably better efficacy of ethyl acetate extract was supported by other previous studies on plant extracts [28–30]. Thus, of the extracts, ethyl acetate extracts might have a better penetration ability of the outer membrane of gram-negative bacteria and disturb cellular function, metabolism, and loss of cellular constituents, leading to their inhibition and death of the bacteria.

Gram-positive bacteria were more susceptible to the extracts than gram-negative bacteria. Many other studies on different medicinal plants also revealed that gram-positive bacteria tend to be more sensitive to the antimicrobial properties of plant extracts than gram-negative bacteria [31–34]. This could be due to gram-negative bacteria having an outer membrane that is composed of high-density lipopolysaccharides that serve as a barrier to many environmental exposures, including antibiotics [35].

In addition, this study confirms that the roots of *I*. *tinctoria* A. Rich also had promising antibacterial activity, especially against *S*. *aureus* and *S*. *epidermis*, which are commonly found in the skin, even though the traditional application is to control fungal infections and toughen the skin [15,16]. Hence, locally dying skin, applying clothes and different materials might prevent infection transmission of Staphylococci (S. *aureus*, MRSA and *S*. *epidermidis*), the most abundant skin-colonizing (biofilm-forming) bacteria and the most important causes of community-associated and hospital-acquired skin infections [36–38].

### Acute toxicity

The evaluation of toxic characteristics is usually a preliminary step in screening medicinal plants for pharmacological activity. However, there is a lack of scientific validation on the toxicity and adverse effects of medicinal plants. Therefore, scientific knowledge towards acute oral toxicity study is needed since it helps to identify the dose that could be used subsequently and to reveal the possible clinical signs elicited by these medicinal plants under investigation. In addition, to increase confidence in medicinal plants or preparation safety for humans, data from toxicity studies should be obtained [39].

The oral acute toxicity study of the tested plant extracts was carried out on albino mice at a single dose of 300, 600,1200,2400,4800 and 9600 mg/kg body weight and was continuously monitored for the first 4 hours, followed by a period of 14 days daily for any toxic effect after the treatment period. Major changes in behavior and mortality were not observed in all groups. However, drowsiness and erection of fur were observed in each mouse of the 4800 and 9600 mg/kg bodyweight treatment groups. These signs disappeared after the 4^th^ hour in almost all of the mice that showed symptoms. The extract seems to be safe at a dose level of 9600 mg/kg, and the LD_50_ is considered to be >9600 mg/kg. According to the Hodge and sterner toxicity classification, the root extract of *I*. *tinctoria* A. Rich is classified as practically nontoxic herbal medicine, as LD_50_ between5000 and 15000 mg/kg is practically nontoxic according to this classification [40].

The body weight of each mouse was carefully weighed on the first day, the 7th day and the day of sacrifice. The body weights of the tested animals in both the control and treated groups increased progressively throughout the study period, although the changes were not statistically significant (p<0.05). Bodyweight changes serve as a sensitive indicator of the general health status of animals [41]. Therefore, the normal increment in body weight and the zero death report could give confidence to the state roots of *I*. *tinctoria* A. Rich did not interfere with the normal metabolism of animals.

## Conclusion

This study provides a scientific basis as the root of *I*.*tinctoria* A. Rich had promising antibacterial activity in an extract-dependent manner in which ethyl acetate extract showed better potency. Gram-positive bacteria, especially *S*.*aureus* and *S*.*epidermidis*, were more susceptible to the extracts than gram-negative bacteria. On the other hand, the acute oral toxicity study of the aqueous extracts of the plant appeared to be safe up to the maximum tested dose that classifies *I*.*tinctoria* A. Rich at least within practically non-toxic category. Further studies, such as exploring the antibacterial novel bioactive molecules, chronic skin and oral toxicity studies, are expected from the scientific community.

## Supporting information

S1 Annexes (DOCX)
