# Supplementary material for: Evaluation of antibacterial and acute oral toxicity of Impatiens tinctoria A. Rich root extracts
Source: PLoS One. 2021 Aug 11;16(8):e0255932. doi: 10.1371/journal.pone.0255932 (PMC8357124; doi:10.1371/journal.pone.0255932)
Supplement: S1 Annexes — (DOCX) [file pone.0255932.s001.docx]

# Annexes

## 1. Standard operating procedures

### 1. 1.SOP of antimicrobial activity

**1.1.1. SOP of screening of antimicrobial effect of extracts by well diffusion method**

**Purpose:** This procedure provides instructions to perform antibacterial hole diffusion assay on natural product.

**Principle:** Reservoir containing the plant extract to be tested is brought into contact with an inoculated medium (e.g. agar) and after incubation; the diameter of clear zone around the reservoir is measured as antimicrobial activity of plant extract.

**Materials:**

| **Supplies and reagents** | **Equipments** |
| --- | --- |
| Organic solvent (solublizer) | Biosafety Cabinet |
| Alcohol | Incubator |
| Detergent | Spectrophotometer |
| Glove | Balance |
| Petridishes | Autoclave |
| Pipette (2, 5, 10 mm) | Sterile cork borers |
| Pipette tips (0-5, 10-200, 1000µl) | Pipette filler |
| Test tube | Micro pipette (0-5, 10-200 µl) |
| Erlenmeyer Flasks | Vortex |
| Measuring Cylinder | Colony counter |
| Test organism | Refrigerator |
| Nutrient broth (Muller-Hinton broth) | Bunsen burner |
| Muller-Hinton agar | Thermometer |
| Standard antibiotics | Deep Freezer |
| Dimethyl Sulfoxide | Borer |
| Distle water |  |
| Test sample (crude extract or synthetic compound) |  |
| Applicator stick |  |
| PH meter |  |

**Sample:** Adequate plant extract that is stable for 3 months with storage of at 4-50c temperature.

**Quality control:** Quality control materials such as standard antibiotics and solublizers should be run with the experiment. Positive control and negative control will run per experiment.

**Procedure and interpretation**

| Step | Action |
| --- | --- |
| 1 | All the test organisms present in the deep freezer will be refreshed by growing with the appropriate media. |
| 2 | Take a loop full of microorganism and standardize by reading the absorbance by spectrophotometer till 0.08-0.1 OD range at 625nm wave length |
| 3 | Take inoculum by a sterile cotton swab and rotate several times and pressed firmly on the inside wall of the tube above the fluid level to remove excess inoculum from the swab. |
| 4 | Streak the swab over the entire sterile agar surface |
| 5 | Perform the assay in triplicate |
| 6 | Punch required numbers of holes using a sterile cork borer ensuring proper distribution of holes (10 cm from the edge of Petri dish and 20 cm from each other holes) in the periphery and one in the center. Agar plugs are removed. |
| 7 | Pour 100 µl in to holes of the test sample dissolved in appropriate solvent into appropriately labeled cups (these are marked at the back of the cup before filling) using a 10-200 sized micro-pipette. Use standard drug in comparison. |
| 8 | Place the plates at room temperature for 2 hr, to allow diffusion of the sample. |
| 9 | Incubate the plate face upwards at 37^O^C for 18-24 hr(for bacteria) at 25 ^O^C for 7 days(for fungus) |
| 10 | The diameter of zones of inhibition is measured to the nearest mm (the cup size also being noted) as positive for antimicrobial activity. No inhibition zone as no any antimicrobial activity. |

**Clinical utility**: For the search of novel antibacterial and antifungal drugs from natural products.

**1.1.2. SOP of Minimum Inhibition Concentration**

**Purpose**: This procedure provides instructions how to perform antibacterial and antifungal agar broth dilution assay of natural products.

**Principle;** A fixed amount of extract mixture is mixed with nutrient broth or Muller-Hinton broth containing bacterium. Colony growth is taken as an indication of microbial density. When no growth takes place, the medium remains clear; when sample is inactive against the germ tested and there is growth.

**Materials:** Appropriate materials in SOP 1.1 will used.
**Sample:** The plant extracts stored at 2-8 ^O^C which is stable for 3 months.

**Quality control:** Quality control materials such as standard antibiotics and solublizers should be run with the experiment.

**Procedure:**

| Step | Action |
| --- | --- |
| 1 | Prepare the extracts by diluting in water (in case of water-soluble samples) or by organic solvents like 5% tween 80 serially in two fold. |
| 2 | Take 2ml of the prepared concentrations and add in to test tubes containing 18 ml of molten agar medium (55^o^C). |
| 3 | Adequately mix and pour into petridishes |
| 4 | Make dry the agar |
| 5 | Take a loop full of microorganism and standardize by reading the absorbance by spectrophotometer till 0.08-0.1 OD range at 625nm wave length |
| 6 | Take 2 microlitre prepared suspension by a micropipette and drop on the prepared extract containing agar surface. |
| 7 | Perform the assay in triplicate |
| 8 | Place the plates at room temperature for 2 hr, to allow diffusion of the sample. |
| 9 | Incubate the plate at 37^O^C for 18- 24 hr(for bacteria) at 25 ^O^C for 7 days(for fungus) |
| 10 | After incubation observe the last petridish with no visible growth of the microorganism is taken to represent the MIC of the test sample which is expressed in mg/ml. |

**Clinical utility**: For the determination of Minimum Inhibitory Concentration of Natural products which will help the search for novel antibacterial drugs from natural products.

**1.1.3. SOP for Minimum Bactericidal/Fungicidal Concentration of the extracts**

**Purpose**: The MBC/MFC assay is performed as an adjunct to the [MIC](https://www.life.umd.edu/classroom/bsci424/LabMaterialsMethods/BrothTubeMIC.htm) and is used to determine the concentration of the extract that is lethal to the target bacteria or fungus in vitro.

**Principle**: This is checking of the lethality effect of the extract by sub culturing all plates not showing visible growth in the MIC test. The inhibition zone in the MIC will be sub cultured and microcidality of the extract will be checked by the absence of growth and if there is growth it assures the inhibition ability of the extract but not lethal.

**Materials**: use appropriate materials on 1.1

**Sample:** Adequate plant extract that is stable for 3 months with storage of at 2-8 ^o^C temperature.

**Quality control:** Quality control materials such as standard antibiotics and solublizers should be run with the experiment. Positive control and negative control will run per experiment.

**Procedure and interpretation:**

1. Inoculums will be taken from each MIC agar plate without visible growth and streak onto appropriate media.
2. Record the dilution of the subculture MIC plates on each plate and incubate as described at MIC part.
3. Then, after incubation, examine the MBC plates for colony growth or lack of growth for each subcultures. No growth indicates that the extract is bactericidal/ fungicidal at that dilution. Growth indicates that the extract is micro-static but not microcidal at that dilution.

**Clinical utility**: For the determination of Minimum Bactericidal Concentration of natural products which will help the search for novel antibacterial drugs from natural products.

### 1.2. Acute oral toxicity test

**Purpose:** This procedure provides instruction how to perform acute oral toxicity test in mice.

**Materials and Reagents: -** Diluting solution

**Supplies:-**Gastric feeding needles (gavages), Glove, Distilled water, Detergent, Alcohol, Beaker 25, 50, 100, 250mL.

**Sample:**

- Sample type: -Plant extracts
- Amount required:-1ml/100g of body weight
- Transport and Storage:- At room temperature
- Stability:- non- applicable

**Principle:** It is based on the principle in which mice were administered orally with different dosages of tested extracts and mortality, signs of toxicity, food and water consumption and change in body weight was observed for 14 days. The information obtained from the observation is useful in choosing doses for repeat-dose studies, providing preliminary identification of target organs of toxicity, and, occasionally, revealing delayed toxicity.

**Procedure:**

| **Step** | **Action** |
| --- | --- |
| **1** | Use Albino mice of either sex, weighting 20-35g. |
| **2** | Acclimatize the animals with the working environment. |
| **3** | Divide the mice’s randomly in to groups based on sex and body weight (five mice in each group). |
| **4** | Withdraw food but not water for 3-4 hours prior to the experiment |
| **5** | Administer the diluting solution for one group as a negative control. |
| **6** | For the other groups administer the plant extract orally via gavage as required dose (Graded dose). |
| **7** | Observe sign of toxicity and mortality for the first four hours and in two hours interval for 24 hours, and daily till 14 days. |

**Procedure Note:**

- The use of vehicle control groups should be considered.
- Animals should be observed for 14 days after administration. All mortalities, clinical signs, time of onset, duration, and reversibility of toxicity should be recorded.

**Calculation: -** Determine LD50

**Result Interpretation**

- Response data and dose level for each animal (i.e. animals showing signs of toxicity including mortality, nature, severity and duration of effects);
- Individual weights of animals at the day of dosing, in weekly intervals thereafter, and at time of death or sacrifice**.**
- Date and time of death if prior to scheduled sacrifice.
- Time course of onset of signs of toxicity and whether these were reversible for

**Clinical Utility**

The information obtained from these studies is useful in choosing doses for repeat-dose studies, providing preliminary identification of target organs of toxicity, and occasionally, revealing delayed toxicity.

## 2. Photographs captured during the study

**2.1. The plant materials in different forms**

**
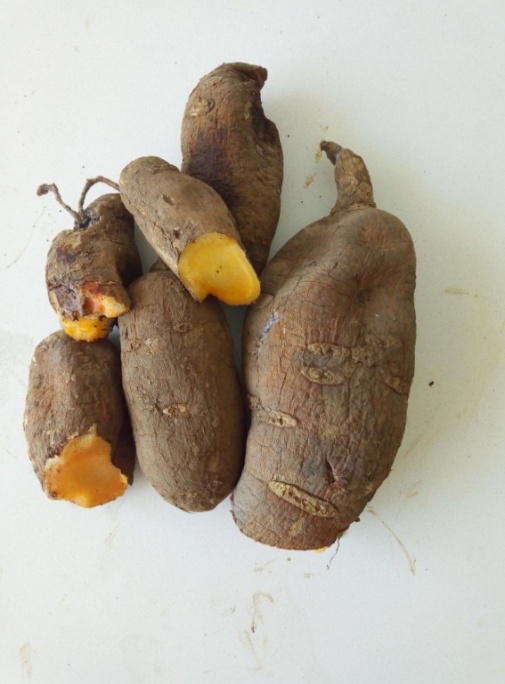

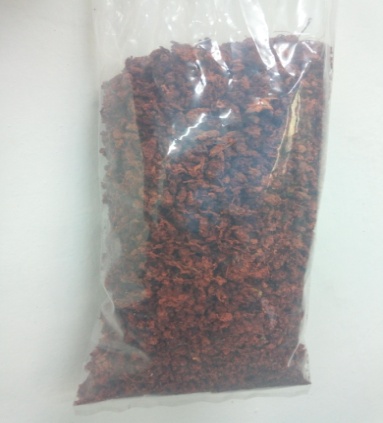

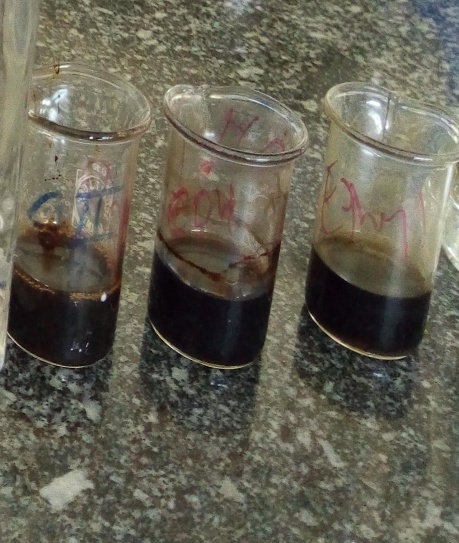
**

**Roots of *I.tinctoria* A. Rich Milled roots Final extracts**

**2.2. Photographs that show some of the procedures during the experiment**

B

A


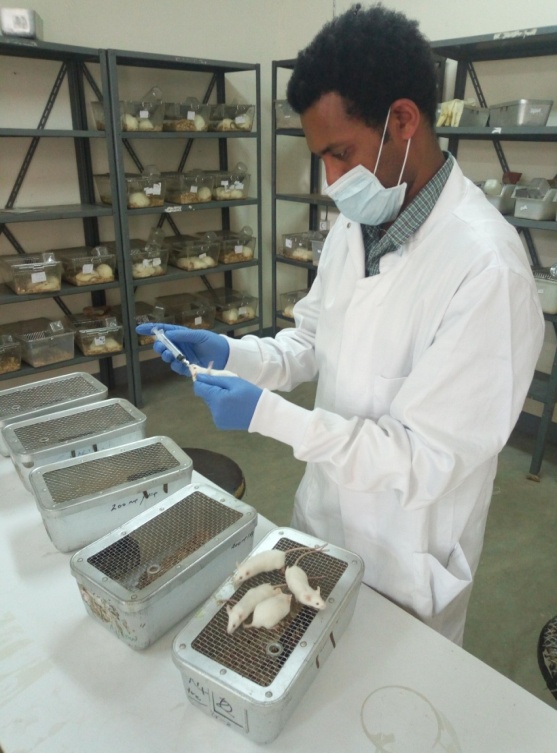

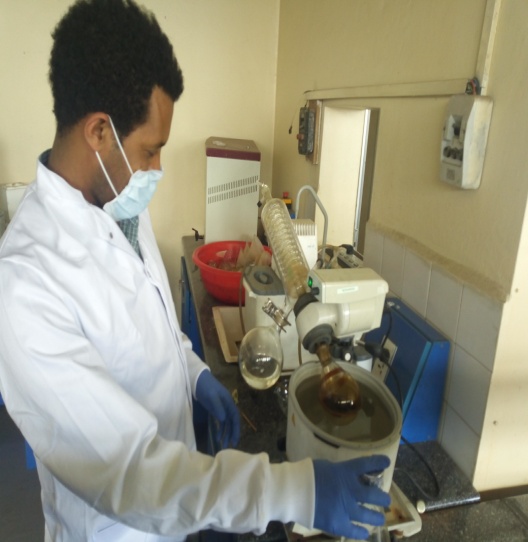


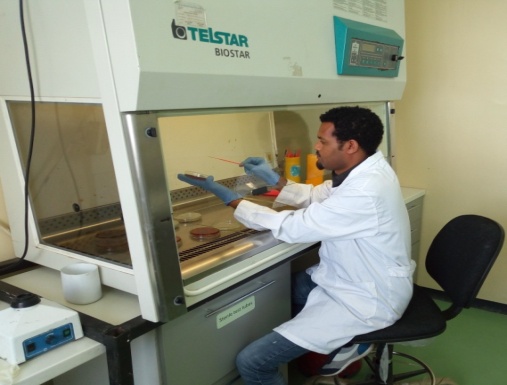


C


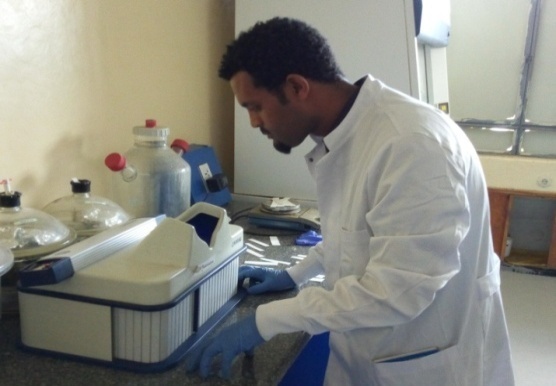

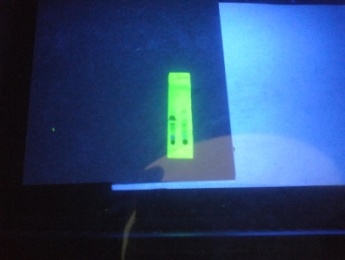


D

Activities during concentrating the macerated filtrate by Rota vapor (A), extracts administration to mice for acute toxicity study (B), antimicrobial assessment(C), TLC reading (D)


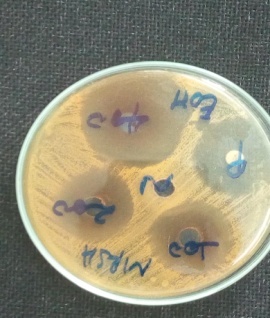
2.3. Some of the photographs that show the inhibition ability of the extracts against tested microorganisms


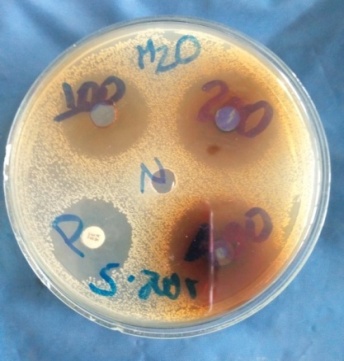

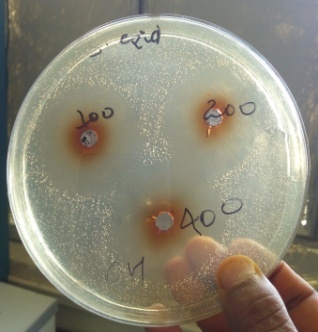


C

B

A


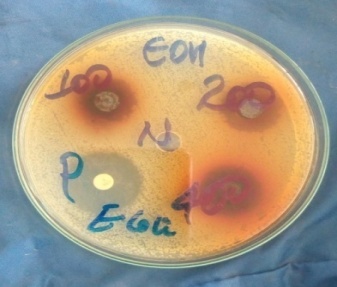


D

Inhibition zone on MRSA (A), *S.aureus* (B), *S.epidermis*(C), *E.coli* (D)

2.5. Photographs of some bacteria MIC plate after incubation


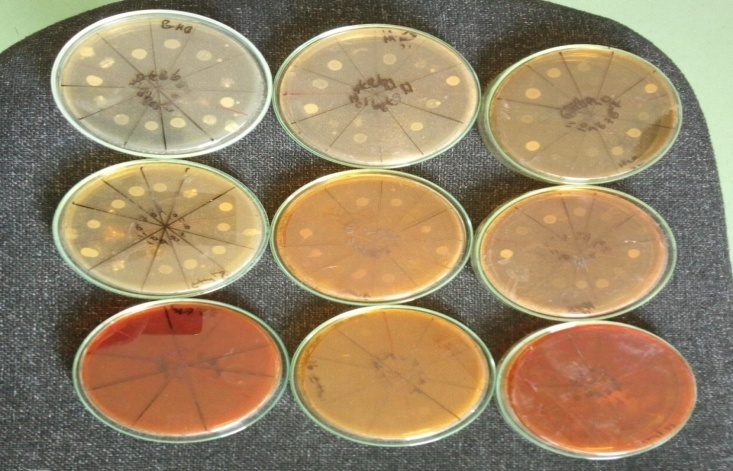


Sample of MIC plates of some bacteria
